# Awareness, factual knowledge, attitudes, and acceptability of human papillomavirus vaccination among adult women in Palestine: a cross-sectional study

**DOI:** 10.3389/fgwh.2026.1841337

**Published:** 2026-07-21

**Authors:** Alhareth M. Amro, Leen M. Safadi, Shahd R. Fahoum, Salahaldeen Deeb, Anas K. Assi, Lamar Aabed, Sadeel Suwan, Rayan Abdalhadi Abdullah, Jomana Sbehat, Raghda Amro, Hazem Agha

**Affiliations:** 1 Faculty of Medicine, Al-Quds University, Jerusalem, Palestine; 2 Faculty of Medicine, An-Najah National University, Nablus, Palestine; 3 Faculty of Medicine, Zagazig University, Zagazig, Sharqia, Egypt; 4 Faculty of Medicine, Palestine Polytechnic University, Hebron, Palestine; 5 Faculty of Public health and Nutrition, Al-Quds University, Jerusalem, Palestine

**Keywords:** awareness, barriers, cervical cancer prevention, factual knowledge, HPV vaccine, human papillomavirus, Palestine, vaccine acceptability

## Abstract

**Background:**

Human papillomavirus (HPV) is a major preventable cause of cervical cancer, yet awareness and uptake of HPV vaccination remain suboptimal in many settings. Evidence regarding women's HPV-related awareness, factual knowledge, and vaccine acceptability in Palestine remains limited. This study assessed awareness, factual knowledge, attitudes, self-reported uptake, willingness, and perceived barriers related to HPV infection and HPV vaccination among adult women in Palestine.

**Methods:**

A cross-sectional online survey was conducted between October 2025 and February 2026 among women aged ≥18 years residing in Palestine. The questionnaire assessed sociodemographic characteristics, HPV and HPV vaccine awareness, factual knowledge, attitudes, vaccine uptake, willingness to receive or recommend vaccination, and perceived barriers. A 12-item factual knowledge score was calculated, and higher factual knowledge was defined as ≥7 correct responses. Descriptive statistics, subgroup analyses, and multivariable logistic regression were performed.

**Results:**

Among 424 adult women included in the final analysis, 220 (51.9%) had heard of HPV and 146 (34.4%) had heard of the HPV vaccine. The mean factual knowledge score was 4.03 ± 2.72 out of 12, and 90 participants (21.2%) had higher factual knowledge. Self-reported HPV vaccine uptake was low (13.0%), whereas willingness to receive the vaccine was higher (55.2%). Lack of awareness (55.8%) and concern about vaccine safety (22.8%) were the most frequently reported barriers among unvaccinated women. In expanded adjusted analyses, prior HPV awareness, prior HPV vaccine awareness, and more supportive HPV-vaccination attitudes were independently associated with higher factual knowledge. Higher factual knowledge and supportive attitudes were associated with self-reported vaccine uptake, while willingness among unvaccinated women was most strongly associated with supportive vaccination attitudes.

**Conclusion:**

Adult women in Palestine demonstrated limited HPV-related awareness and low factual knowledge, together with low self-reported HPV vaccine uptake. The gap between willingness and uptake, combined with the predominance of awareness and safety concerns as reported barriers, suggests a need for culturally appropriate education, provider-led counseling, and improved access to reliable HPV vaccine information. Findings should be interpreted cautiously given the cross-sectional design and online convenience sampling.

## Introduction

Cervical cancer prevention is now framed globally within the World Health Organization cervical cancer elimination strategy, which emphasizes HPV vaccination, cervical screening, and treatment of cervical precancer and cancer as complementary pillars of prevention. The strategy sets a 2030 target that 90% of girls should be fully vaccinated with HPV vaccine by age 15 years (1). Current WHO guidance also emphasizes the role of HPV vaccination in preventing cervical cancer and other HPV-related diseases (2).

Human papillomavirus (HPV) is the most common sexually transmitted viral infection worldwide and a well-established cause of cervical cancer, as well as other anogenital and oropharyngeal malignancies. Although most HPV infections are transient and resolve spontaneously, persistent infection with high-risk genotypes can lead to precancerous lesions and invasive cancer. HPV-related disease therefore remains an important, yet largely preventable, public health burden (3).

Despite the demonstrated effectiveness of HPV vaccines, uptake remains suboptimal in many settings. Low awareness of HPV, misconceptions regarding vaccine safety and eligibility, sociocultural sensitivities surrounding sexually transmitted infections, and limited healthcare-provider recommendation may all reduce vaccine acceptance. In such contexts, poor uptake may reflect inadequate knowledge and limited access to reliable health information rather than outright refusal (4). Assessing awareness, factual knowledge, attitudes, and vaccine acceptability is therefore essential for informing targeted prevention strategies. Women's understanding of HPV transmission, its association with cervical cancer, and the benefits of vaccination may substantially influence their willingness to be vaccinated or to recommend vaccination to others. Identifying these factors within specific populations is important for developing effective educational interventions and informing future policy (5).

In Palestine, evidence regarding women's awareness of HPV infection and perceptions of HPV vaccination remains limited. This gap restricts efforts to design contextually appropriate educational initiatives and to support broader HPV prevention strategies. Accordingly, this study aimed to assess awareness, factual knowledge, attitudes, and acceptability regarding HPV infection and HPV vaccination among adult women in Palestine, in addition to evaluating self-reported vaccine uptake, perceived barriers to vaccination, and sociodemographic factors associated with better factual knowledge.

## Methods

### Study design and setting

This study employed a cross-sectional, questionnaire-based design to assess awareness, factual knowledge, attitudes, and acceptability regarding human papillomavirus (HPV) infection and HPV vaccination among women in Palestine. Data were collected over a 5-month period, from October 2025 to February 2026, using a self-administered online survey.

### Study population and eligibility criteria

The study was designed to assess HPV-related awareness and HPV vaccine acceptability among women. However, because the survey link was distributed through open online platforms, access could not be technically restricted by sex at the point of link sharing. Sex and age were therefore collected as screening variables. Male respondents and respondents aged <18 years were excluded before analysis according to the prespecified eligibility criteria. The study was not designed to analyze male respondents, and the small number of male submissions was treated as ineligible rather than as a comparison group.

### Sample size calculation

The minimum required sample size was calculated for a single population proportion using the formula *n* = *Z*^2^*p* (1 − *p*)/*d*^2^. Because no precise prior estimate was assumed for HPV vaccine awareness in the target population, *p* was set at 0.50 to provide the most conservative estimate. Using a 95% confidence level (*Z* = 1.96) and a 5% margin of error (*d* = 0.05), the minimum required sample size was 384 participants. The final analytic sample of 424 adult women exceeded this requirement. Under the same assumptions, the achieved sample size provides a maximum approximate margin of error of ±4.8% for proportions near 50%.

### Study instrument

Data were collected using a structured questionnaire adapted from a previously published study investigating HPV awareness and HPV vaccination among women in the Medina region (6). The adapted questionnaire included sections on sociodemographic characteristics, prior awareness of HPV and HPV vaccination, factual knowledge, vaccine uptake and acceptability, attitudes toward HPV vaccination, and perceived barriers. The questionnaire was reviewed by the study team for content relevance, clarity, and suitability for the Palestinian context. Because a fully locally validated Palestinian HPV vaccine knowledge instrument was not available, internal consistency was assessed in the present analytic sample.

The 12-item factual knowledge scale demonstrated acceptable internal consistency, with a Kuder–Richardson 20/Cronbach's alpha coefficient of 0.761. Attitude items were primarily analyzed descriptively because they addressed related but not fully identical domains, including vaccine confidence, perceived risk, safety concerns, school-based education, and policy support. For exploratory regression analyses, a 5-item supportive HPV-vaccination attitude index was created using items assessing perceived vaccine effectiveness, acceptability of side effects, preference for vaccination before sexual activity, support for school-based HPV education, and support for inclusion of HPV vaccination in the national immunization program. This index demonstrated good internal consistency in the present sample, with Cronbach's alpha = 0.803. Higher scores indicated more supportive attitudes toward HPV vaccination.

### Variable definitions and outcome measures

The primary analytic outcome was factual knowledge regarding HPV infection and HPV vaccination. A 12-item factual knowledge score was constructed using objectively scoreable items covering sexual transmission, spontaneous clearance, cervical cancer causation, vaccine effectiveness, prevention of cervical cancer, vaccine availability, possible side effects, the misconception that vaccination can cause HPV infection, reduction in abnormal Pap smear changes, screening requirements before vaccination for women and men, and the recommended target group for vaccination.

Each correct response was assigned a score of 1, whereas incorrect responses, “I don't know,” and nonstandard responses were assigned a score of 0. The total score therefore ranged from 0 to 12, with higher scores indicating better factual knowledge. The total factual knowledge score was analyzed as a continuous variable and summarized using the mean, standard deviation, median, and interquartile range. For categorical interpretation, participants were classified as having higher factual knowledge if they achieved at least 7 correct responses out of 12. This threshold was selected *a priori* because 6 correct responses represent exactly 50%, whereas 7 correct responses represent more than 50% correct answers. To reduce reliance on a single dichotomous cutoff, item-level performance and the continuous knowledge score were also reported.

Items related to prior awareness of HPV or the HPV vaccine, information sources, self-reported vaccination schedule knowledge, previous vaccination, willingness to vaccinate, recommendation to others, attitudinal statements, and barriers to vaccination were analyzed separately and were not included in the factual knowledge score.

Attitude items were measured on a five-point Likert scale ranging from strongly disagree to strongly agree. For descriptive analysis, responses were collapsed into three categories: agree/strongly agree, neutral, and disagree/strongly disagree. The barriers item allowed multiple responses; therefore, percentages for barrier categories were calculated using the number of unvaccinated women as the denominator and were not mutually exclusive.

### Data collection and management

A convenience, open online sampling approach was used. The survey link was distributed electronically through commonly used online platforms, including WhatsApp, Facebook, Instagram, university groups, community networks. The invitation included a brief description of the study objectives, eligibility criteria, voluntary nature of participation, and anonymity of responses. Participants were also allowed to share the survey link with potentially eligible women, resulting in a snowball component to recruitment. Because the survey was distributed through open online channels, the number of individuals who received or viewed the invitation could not be determined; therefore, a conventional response rate could not be calculated. Instead, we report the number of submitted questionnaires, exclusions, and final analytic sample.

### Statistical analysis

Data were analyzed using IBM SPSS Statistics version 25. Categorical variables were summarized as frequencies and percentages. Continuous variables, including the factual knowledge score, were summarized using the mean and standard deviation, and where appropriate, the median and interquartile range. Descriptive analyses were performed for individual knowledge items, awareness measures, vaccine uptake and acceptability variables, attitude statements, and barriers to vaccination.

Bivariable subgroup analyses were conducted to compare HPV vaccine awareness, higher factual knowledge, HPV vaccine uptake, and willingness to receive the vaccine across sociodemographic categories using the chi-square test or Fisher's exact test, as appropriate. Multivariable logistic regression was then used to identify factors independently associated with three outcomes: higher factual knowledge, self-reported HPV vaccine uptake, and willingness to receive HPV vaccination among unvaccinated women. The expanded model for higher factual knowledge included age group, marital status, educational level, employment status, monthly family income, prior awareness of HPV, prior awareness of the HPV vaccine, and the supportive HPV-vaccination attitude score. Because self-reported vaccine uptake was reported by a smaller number of participants, models for vaccine uptake and willingness used collapsed sociodemographic categories to reduce model overfitting. Results were reported as adjusted odds ratios (aORs) with 95% confidence intervals (CIs). All statistical tests were two-sided, and *p* < 0.05 was considered statistically significant.

### Ethics approval and consent to participate

The study was approved by the Institutional Review Board Committee of Al-Quds University. Participation was voluntary, and electronic informed consent was obtained from all participants before questionnaire completion. The questionnaire was anonymous, no personally identifying information was collected, and all data were handled confidentially and used solely for research purposes.

## Results

**Figure 1:**
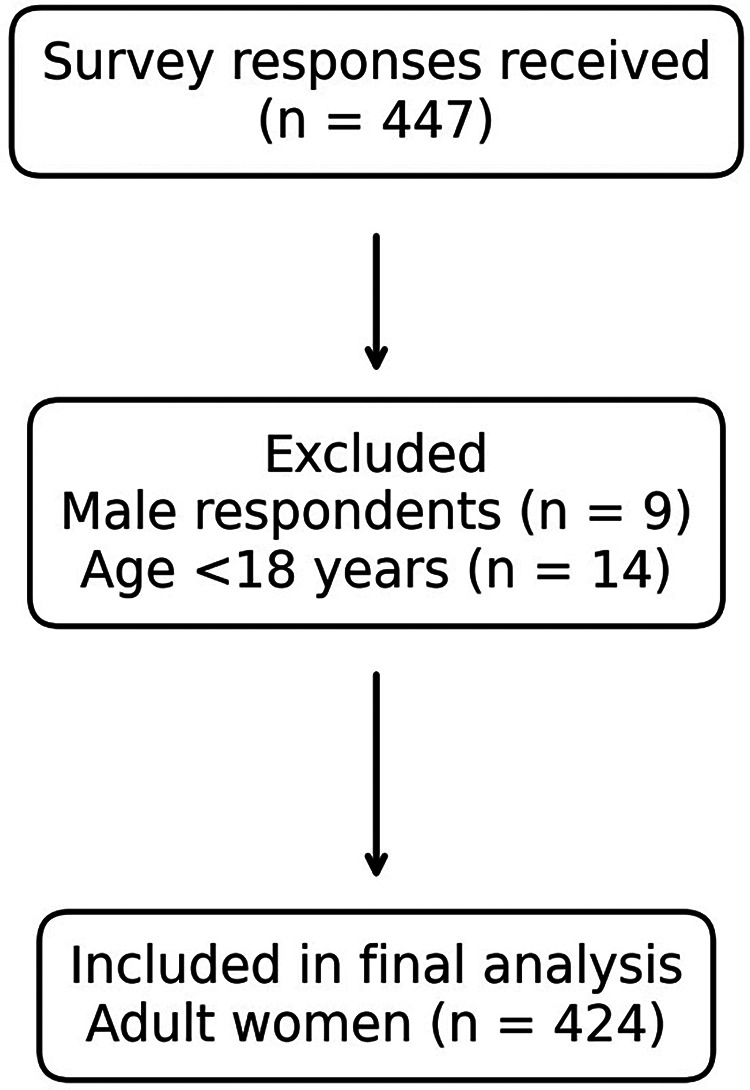
Participant flow diagram.

A total of 447 questionnaire responses were received. After exclusion of 9 male respondents and 14 respondents aged younger than 18 years, 424 adult women were included in the final analysis (Figure 1). The study population was predominantly young, with 274 participants (64.6%) aged 18–24 years. Most respondents were single (65.8%), had higher education (88.7%), and were unemployed (55.2%) (Table 1).

**Table 1:** Demographic characteristics of the study participants (*N* = 424).

Variable	Category	*n*	%
Age	18–24 years	274	64.6
	25–34 years	55	13.0
	35–44 years	38	9.0
	45–54 years	45	10.6
	55–64 years	10	2.4
	65+ years	2	0.5
Marital status	Single	279	65.8
	Married	130	30.7
	Divorced	11	2.6
	Widowed	4	0.9
Educational level	<High school	4	0.9
	Secondary school	44	10.4
	Higher education	376	88.7
Occupational status	Government sector	83	19.6
	Private sector	56	13.2
	Self-employed	43	10.1
	Unemployed	234	55.2
	Retired	8	1.9
Monthly family income	Low	39	9.2
	Average	232	54.7
	High	153	36.1

Percentages may not sum to 100 because of rounding.

Overall awareness of HPV and its vaccine was limited. Only 220 women (51.9%) reported that they had heard of HPV, and 146 (34.4%) had heard of the HPV vaccine. The mean factual knowledge score was 4.03 ± 2.72 out of 12 items, with a median of 4 (IQR 2–6). Using a prespecified threshold of at least 7 correct responses, 90 participants (21.2%) were classified as having good factual knowledge (Table 2).

**Table 2:** Awareness, uptake, vaccine acceptability, and factual knowledge summary (*N* = 424).

Outcome	Value
Heard of HPV	220 (51.9)
Heard of HPV vaccine	146 (34.4)
Received HPV vaccine	55 (13.0)
Willing to receive HPV vaccine	234 (55.2)
Would recommend vaccine for child/adolescent (9–12 years)	144 (34.0)
Would recommend vaccine for friend/relative	261 (61.6)
Factual knowledge score, mean ± SD	4.03 ± 2.72
Factual knowledge score, median (IQR)	4 (2–6)
Achieved ≥6/12 correct responses	134 (31.6)
Higher factual knowledge (≥7/12 correct responses)	90 (21.2)
Achieved ≥8/12 correct responses	50 (11.8)

Higher factual knowledge was defined *a priori* as at least 7 correct responses on the 12-item factual knowledge scale. Score-distribution thresholds are shown to reduce overreliance on a single dichotomous cutoff.

**Figure 2:**
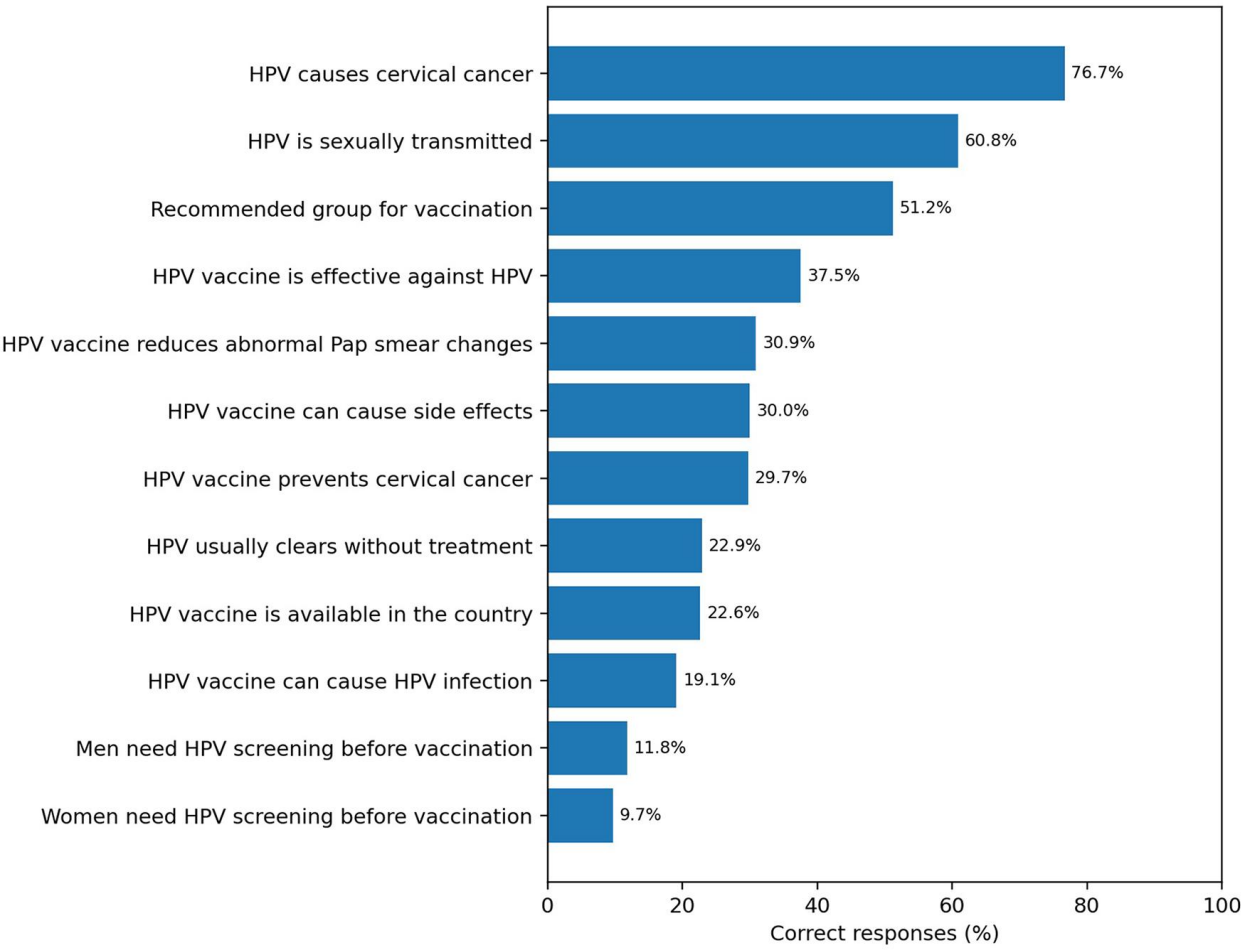
Primary sources of HPV vaccine information among respondents who had heard of the HPV vaccine (*n* = 146).

Item-level performance showed marked variation across the factual knowledge items (Table 3, Figure 2). The highest correct response rate was observed for the statement that HPV causes cervical cancer (325/424, 76.7%), followed by the item that HPV is sexually transmitted (258/424, 60.8%). In contrast, fewer than one-third of respondents correctly identified that HPV usually clears without treatment (22.9%), that the vaccine is available in the country (22.6%), that the vaccine does not cause HPV infection (19.1%), and that screening before vaccination is not required for women (9.7%) or men (11.8%).

**Table 3:** Item-level responses for the 12-item factual knowledge scale (*N* = 424).

Item	Correct answer	Correct *n* (%)	Incorrect *n* (%)	Don't know/other *n* (%)
HPV is sexually transmitted	Yes	258 (60.8)	166 (39.2)	0 (0.0)
HPV usually clears without treatment	Yes	97 (22.9)	327 (77.1)	0 (0.0)
HPV causes cervical cancer	Yes	325 (76.7)	99 (23.3)	0 (0.0)
HPV vaccine is effective against HPV	Yes	159 (37.5)	33 (7.8)	232 (54.7)
HPV vaccine prevents cervical cancer	Yes	126 (29.7)	48 (11.3)	250 (59.0)
HPV vaccine is available in the country	Yes	96 (22.6)	35 (8.3)	293 (69.1)
HPV vaccine can cause side effects	Yes	127 (30.0)	46 (10.8)	251 (59.2)
HPV vaccine can cause HPV infection	No	81 (19.1)	83 (19.6)	260 (61.3)
HPV vaccine reduces abnormal Pap smear changes	Yes	131 (30.9)	37 (8.7)	256 (60.4)
Women need HPV screening before vaccination	No	41 (9.7)	203 (47.9)	180 (42.5)
Men need HPV screening before vaccination	No	50 (11.8)	152 (35.8)	222 (52.4)
Recommended group for vaccination	Both genders	217 (51.2)	82 (19.3)	125 (29.5)

“Don't know/other” includes “I don't know” and nonstandard responses.

In sensitivity descriptions of the score distribution, 134 participants (31.6%) achieved at least 6 correct responses, 90 (21.2%) achieved at least 7 correct responses, and 50 (11.8%) achieved at least 8 correct responses. The ≥7/12 threshold was therefore used only as a pragmatic categorical indicator of higher factual knowledge, while the continuous score and item-level responses were retained for interpretation.

In subgroup analyses, prior awareness of the HPV vaccine differed significantly by age group, marital status, educational level, and employment status. HPV vaccine awareness was reported by 29.2% of women aged 18–24 years, 47.3% of those aged 25–34 years, and 42.1% of those aged ≥35 years (*p* = 0.007). Awareness was also higher among ever-married women than single women (42.1% vs. 30.5%, *p* = 0.017), among women with higher education compared with secondary education or less (36.7% vs. 16.7%, *p* = 0.006), and among employed compared with not-employed women (40.1% vs. 30.2%, *p* = 0.033). Higher factual knowledge did not differ significantly across most sociodemographic groups, although a non-significant increasing pattern was observed across income categories (low income 12.8%, average income 19.0%, high income 26.8%; *p* = 0.074). Vaccine uptake and willingness to receive the vaccine did not show consistent statistically significant differences across sociodemographic subgroups.

**Figure 3:**
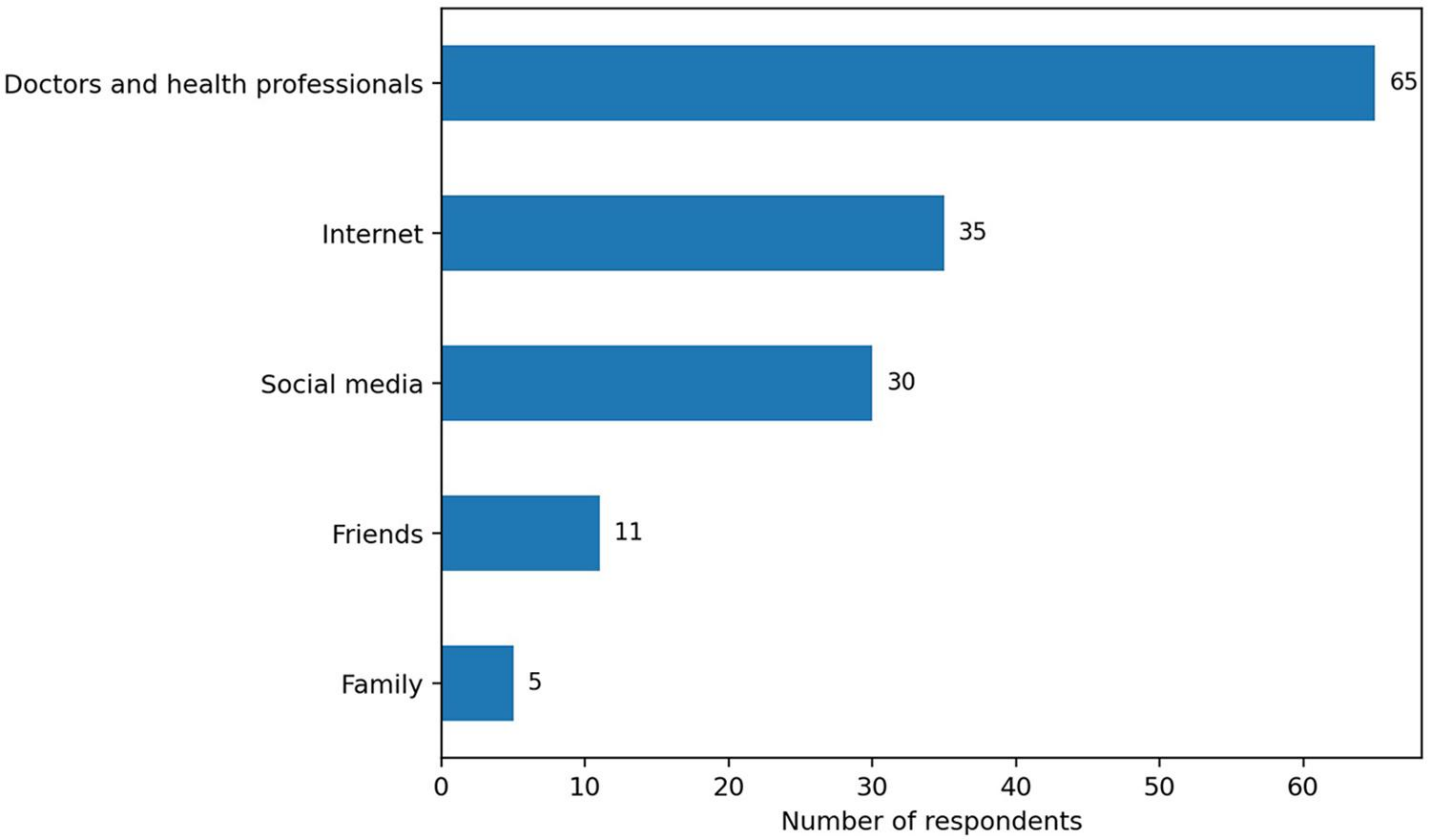
Correct response rates across the 12-item factual knowledge scale (*n* = 424).

Vaccination uptake remained low: 55 women (13.0%) reported having received the HPV vaccine. However, stated vaccine acceptability was more favorable than actual uptake. A total of 234 respondents (55.2%) reported willingness to receive the vaccine, 144 (34.0%) would recommend it for a child or adolescent aged 9–12 years, and 261 (61.6%) would recommend it for a friend or relative (Table 2). Among respondents who had heard of the HPV vaccine, doctors and health professionals were the most frequently reported information source (44.5%), followed by the internet (24.0%), social media (20.5%), friends (7.5%), and family (3.4%) (Figure 3).

Attitudinal responses revealed substantial uncertainty, with neutral responses being common across most statements (Table 4). Nevertheless, support was strongest for school-based HPV education, which was endorsed by 294 respondents (69.3%), and for inclusion of the HPV vaccine in the national immunization program, which was endorsed by 256 respondents (60.4%). In addition, 207 participants (48.8%) agreed that vaccination is preferable before the onset of sexual activity. Notably, 295 women (69.6%) agreed that they would need more information about HPV and its vaccine before deciding to vaccinate.

**Table 4:** Attitudes toward HPV vaccination (*N* = 424).

Statement	Agree/strongly agree *n* (%)	Neutral *n* (%)	Disagree/strongly disagree *n* (%)
Vaccine is effective in preventing cervical cancer	213 (50.2)	180 (42.5)	31 (7.3)
Would take the vaccine because of perceived risk	154 (36.3)	176 (41.5)	94 (22.2)
Having one sex partner protects against HPV	179 (42.2)	180 (42.5)	65 (15.3)
Vaccination is not necessary	92 (21.7)	201 (47.4)	131 (30.9)
Potential side effects are acceptable	162 (38.2)	210 (49.5)	52 (12.3)
Vaccination is better before sexual activity	207 (48.8)	177 (41.7)	40 (9.4)
More information is needed before vaccination	295 (69.6)	109 (25.7)	20 (4.7)
Vaccine may have long negative effects	116 (27.4)	235 (55.4)	73 (17.2)
Only sexually active women should be vaccinated	111 (26.2)	186 (43.9)	127 (30.0)
Parents would not allow vaccination	67 (15.8)	217 (51.2)	140 (33.0)
HPV education should be implemented in schools	294 (69.3)	105 (24.8)	25 (5.9)
Vaccine should be included in the national immunization program	256 (60.4)	145 (34.2)	23 (5.4)

Responses were collapsed from a five-point Likert scale for descriptive reporting.

In the expanded multivariable model for higher factual knowledge, sociodemographic variables were not independently associated with higher factual knowledge. However, prior awareness of HPV (aOR 2.96, 95% CI 1.28–6.86; *p* = 0.012), prior awareness of the HPV vaccine (aOR 5.28, 95% CI 2.56–10.91; *p* < 0.001), and higher supportive HPV-vaccination attitude score (aOR 3.70 per 1-point increase, 95% CI 2.25–6.08; *p* < 0.001) were independently associated with higher factual knowledge (Table 5).

**Table 5:** Multivariable logistic regression for factors associated with higher factual knowledge (*N* = 424).

Predictor	Adjusted OR	95% CI	*p* value
Age 25–34 vs. 18–24	0.60	0.22–1.62	0.311
Age ≥35 vs. 18–24	0.85	0.30–2.42	0.762
Ever married vs. single	1.11	0.46–2.72	0.812
Higher education vs. secondary or less	0.51	0.19–1.40	0.193
Employed vs. not employed	1.10	0.57–2.13	0.776
Average income vs. low	1.46	0.45–4.75	0.532
High income vs. low	1.60	0.48–5.33	0.443
Heard of HPV	2.96	1.28–6.86	0.012
Heard of HPV vaccine	5.28	2.56–10.91	<0.001
Supportive attitude score, per 1-point increase	3.70	2.25–6.08	<0.001

Higher factual knowledge was defined as ≥7 correct responses on the 12-item factual knowledge scale. The supportive attitude score was calculated as the mean of five Likert-type items assessing perceived vaccine effectiveness, acceptability of side effects, preference for vaccination before sexual activity, support for school-based HPV education, and support for inclusion of HPV vaccination in the national immunization program. The 12-item factual knowledge scale demonstrated acceptable internal consistency (KR-20/Cronbach's alpha = 0.761), and the supportive attitude index demonstrated good internal consistency (Cronbach's alpha = 0.803).

**Figure 4:**
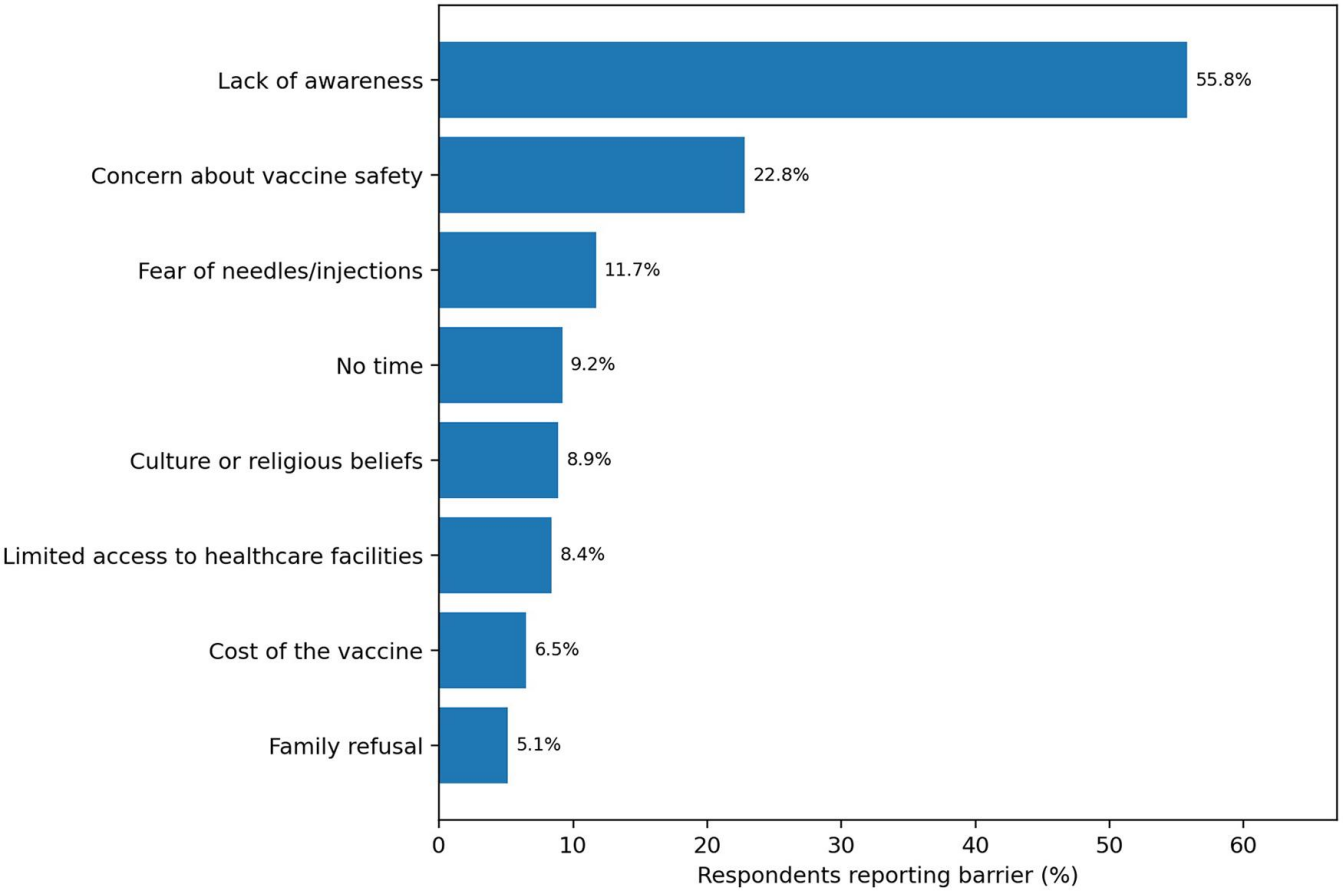
Reported barriers to HPV vaccination among unvaccinated women (*n* = 369). Percentages are based on multiple-response data and are therefore not mutually exclusive.

Among the 369 women who had not been vaccinated, the most frequently reported barrier was lack of awareness about HPV and the vaccine, cited by 206 respondents (55.8%). Concern about vaccine safety was reported by 84 women (22.8%), followed by fear of needles or injections (11.7%), lack of time (9.2%), culture or religious beliefs (8.9%), limited access to healthcare facilities (8.4%), cost of the vaccine (6.5%), and family refusal (5.1%) (Table 6, Figure 4). Because the barriers item permitted multiple selections, percentages are not mutually exclusive.

**Table 6:** Reported barriers to HPV vaccination among unvaccinated women (*n* = 369).

Barrier among unvaccinated women	*n*	%
Lack of awareness	206	55.8
Concern about vaccine safety	84	22.8
Fear of needles/injections	43	11.7
No time	34	9.2
Culture or religious beliefs	33	8.9
Limited access to healthcare facilities	31	8.4
Cost of the vaccine	24	6.5
Family refusal	19	5.1
Other	133	36.0

Multiple responses were allowed; percentages are therefore not mutually exclusive.

In the exploratory model for self-reported HPV vaccine uptake, higher factual knowledge was associated with greater odds of having received the HPV vaccine (aOR 3.27, 95% CI 1.59–6.74; *p* = 0.001). A higher supportive HPV-vaccination attitude score was also associated with vaccine uptake (aOR 1.92 per 1-point increase, 95% CI 1.14–3.25; *p* = 0.014) (Table 7). Prior awareness of the HPV vaccine showed a borderline association with uptake (aOR 2.09, 95% CI 0.98–4.45; *p* = 0.056).

**Table 7:** Exploratory multivariable logistic regression models for HPV vaccine uptake and willingness to receive HPV vaccination.

Outcome	Predictor	Adjusted OR	95% CI	*p* value
HPV vaccine uptake	Age ≥25 years	0.82	0.32–2.08	0.671
	Ever married	0.47	0.19–1.17	0.105
	Higher education	0.84	0.29–2.46	0.746
	Employed	1.79	0.89–3.60	0.101
	High income	1.28	0.68–2.42	0.446
	Heard of HPV vaccine	2.09	0.98–4.45	0.056
	Higher factual knowledge	3.27	1.59–6.74	0.001
	Supportive attitude score, per 1-point increase	1.92	1.14–3.25	0.014
Willingness among unvaccinated women	Age ≥25 years	0.64	0.30–1.35	0.242
	Ever married	0.99	0.50–1.95	0.967
	Higher education	0.89	0.43–1.86	0.762
	Employed	1.46	0.86–2.49	0.163
	High income	0.61	0.39–0.98	0.041
	Heard of HPV vaccine	0.86	0.48–1.52	0.602
	Higher factual knowledge	0.85	0.42–1.71	0.655
	Supportive attitude score, per 1-point increase	3.16	2.14–4.67	<0.001

Models used collapsed sociodemographic categories to reduce overfitting. Willingness was analyzed among unvaccinated women only. These models are exploratory and should be interpreted cautiously because of the cross-sectional design and limited number of vaccinated participants.

Among unvaccinated women, willingness to receive HPV vaccination was strongly associated with the supportive HPV-vaccination attitude score (aOR 3.16 per 1-point increase, 95% CI 2.14–4.67; *p* < 0.001). High income was inversely associated with willingness in this model (aOR 0.61, 95% CI 0.39–0.98; *p* = 0.041), although this finding should be interpreted cautiously given the exploratory nature of the analysis. Prior HPV vaccine awareness and higher factual knowledge were not independently associated with willingness after adjustment.

## Discussion

This study found limited HPV-related awareness and low factual knowledge among adult women in Palestine, despite moderate willingness to receive HPV vaccination. Only 34.4% of participants had heard of the HPV vaccine, 13.0% reported prior vaccination, and 21.2% met the prespecified threshold for higher factual knowledge. In expanded adjusted analyses, prior awareness of HPV, prior awareness of the HPV vaccine, and more supportive HPV-vaccination attitudes were independently associated with higher factual knowledge. In addition, higher factual knowledge and supportive attitudes were associated with self-reported vaccine uptake, while willingness among unvaccinated women was most strongly associated with supportive vaccination attitudes. These findings suggest that the gap between vaccine acceptability and vaccine uptake may be related to informational uncertainty, confidence in vaccine safety, and access to trusted vaccine guidance, although causal inference is not possible because of the cross-sectional design (7, 8).

The attitudinal findings further support the interpretation that uncertainty, rather than firm resistance, may be a major determinant of HPV vaccine hesitancy in this population. A substantial proportion of participants reported needing additional information before making a vaccination decision, highlighting the importance of informed counseling and accessible health education. Furthermore, the predominance of lack of awareness and safety concerns as reported barriers suggests that misconceptions and informational deficits remain central obstacles to vaccine uptake. Although social and cultural factors may also influence vaccination decisions, the present findings indicate that inadequate knowledge and limited confidence in vaccine safety may represent more immediate and modifiable barriers. These results underscore the need for clear, culturally sensitive, and evidence-based communication strategies that address common misconceptions and strengthen public trust in HPV vaccination.

A particularly relevant comparison is with the cross-sectional study conducted in the Medina region, as the questionnaire used in the present study was adapted from that instrument (6). This methodological similarity enhances the comparability of the two studies. In the Medina study, 59.4% of participants had heard of HPV and 54.4% had heard of the HPV vaccine, whereas the corresponding proportions in the present study were lower at 51.9% and 34.4%, respectively. Similarly, willingness to receive the vaccine was higher in the Medina cohort (63.9%) than in the present study (55.2%). By contrast, self-reported vaccine uptake was higher in the present study (13.0%) than in the Medina study (3.1%). Both studies nevertheless converged on the same central pattern: awareness and knowledge remained suboptimal, while lack of awareness was identified as a major barrier to vaccination. This consistency is particularly important given the shared questionnaire framework, and it supports the interpretation that insufficient awareness and incomplete understanding remain key obstacles to HPV vaccine uptake in the region.

The findings of the present study are also comparable to those reported in the Saudi nationwide study (9). In that study, 49.4% of women were aware of HPV and 38.0% were aware of the HPV vaccine, figures that are broadly similar to those observed in the present study. However, willingness to receive the vaccine was higher in the Saudi study (67.6%) than in the present study (55.2%), whereas vaccine uptake was markedly lower (1.4% vs. 13.0%). This discrepancy should be interpreted with caution, as willingness in the Saudi study was assessed in the context of receiving the vaccine free of charge, which may have increased stated acceptability. Importantly, that study also demonstrated a substantial rise in awareness following a national awareness campaign, suggesting that public health messaging can meaningfully improve HPV-related awareness. This observation reinforces the implications of the present findings, namely that low uptake may reflect remediable informational and structural barriers rather than fixed resistance to vaccination.

A further comparison can be made with the Turkish study (10), which assessed women living in a rural setting. In that cohort, only 9.8% of participants had heard of HPV, a proportion markedly lower than that observed in the present study. Despite this very limited awareness, 56.6% of women reported willingness to receive the vaccine if it were offered free of charge, which is remarkably close to the willingness reported in the present study (55.2%). The Turkish study also found similarly favorable attitudes toward vaccinating children under a free-vaccine scenario. Taken together, these findings suggest that low awareness does not necessarily equate to low acceptability. Rather, they support the interpretation that women may remain receptive to HPV vaccination even when baseline knowledge is limited, provided that financial, informational, and healthcare-access barriers are adequately addressed.

The findings of the present study carry important implications for HPV prevention strategies in Palestine. The combination of low awareness, poor factual knowledge, and low vaccine uptake, together with comparatively higher willingness to receive the vaccine, indicates that substantial unmet potential exists for improving HPV vaccine acceptance through targeted public health intervention (11). In particular, the observation that many participants reported needing additional information before making a vaccination decision suggests that educational deficits remain a central and modifiable obstacle to vaccine uptake (12).

When considered alongside studies from more distant settings, the present findings suggest that limited HPV knowledge and vaccine uncertainty are global challenges, although their drivers may differ by health-system context. In high-income settings, HPV vaccination may be available through routine services, yet incomplete awareness, safety concerns, and inconsistent provider recommendation can still limit acceptance and uptake (13). In contrast, in many low- and middle-income settings, knowledge gaps may be compounded by affordability, limited access to vaccination services, and lack of organized school-based or national vaccination delivery. Studies from Bangladesh and from Pakistan/Afghanistan have similarly reported insufficient HPV-related knowledge and a need for targeted education, while studies from high-income settings such as Italy and the United States highlight the continuing importance of vaccine confidence and healthcare-provider communication even where vaccines are more widely available. These comparisons indicate that Palestinian HPV prevention efforts should combine public education with practical access strategies and provider-led counseling rather than relying on awareness campaigns alone (14).

The present findings also align with evidence from settings beyond the Middle East. Studies from high-income countries continue to show that HPV vaccine hesitancy, incomplete awareness, and safety concerns can persist despite vaccine availability and established immunization recommendations. In Italy, adult HPV vaccine acceptance was influenced by knowledge, confidence, and healthcare-provider communication, emphasizing that vaccine availability alone may be insufficient without targeted education and trust-building (8). Recent evidence from the United States has similarly shown that public awareness of HPV, HPV vaccination, and HPV-related cancers remains uneven across populations and regions (15). In low- and middle-income settings outside the region, studies among women in Bangladesh, Pakistan, and Afghanistan have also reported gaps in HPV knowledge, safety concerns, and a need for accessible educational interventions (5, 16). These comparisons suggest that knowledge gaps and confidence-related barriers are not unique to Palestine, although local policy, access, cost, and sociocultural context shape how these barriers affect vaccine uptake.

These findings underscore the need for culturally appropriate and evidence-based educational initiatives aimed at improving women's understanding of HPV infection, its link to cervical cancer, and the preventive value of vaccination (16). Such interventions should not only increase general awareness, but also address specific misconceptions related to vaccine safety, eligibility, and timing of administration. Given that healthcare professionals are often regarded as trusted sources of health information, strengthening provider-based counseling may be especially important for improving confidence in HPV vaccination and facilitating informed decision-making (17).

These findings support a combined strategy that addresses knowledge, confidence, and access. Educational messages should clearly explain HPV transmission, the relationship between HPV and cervical cancer, vaccine safety, recommended timing, and eligibility (18). Healthcare providers should be supported to deliver consistent counseling, as provider recommendation is often a trusted source of vaccine information. At the policy level, school-based education, reproductive-health counseling, and community cancer-awareness programs may provide feasible channels for HPV prevention messaging. Future studies using representative sampling and qualitative methods are needed to better understand geographic, cultural, and health-system barriers and to evaluate interventions designed to improve informed HPV vaccine uptake (19).

These findings should be considered within the broader cervical cancer prevention context. The World Health Organization's cervical cancer elimination strategy emphasizes HPV vaccination, cervical screening, and treatment of precancerous and invasive disease as complementary pillars of prevention, with a 2030 target of 90% of girls fully vaccinated with HPV vaccine by age 15 years. In settings where HPV vaccination is not fully integrated into routine publicly funded immunization pathways, willingness to vaccinate may not translate into uptake unless it is supported by affordable access, clear eligibility guidance, provider recommendation, and organized delivery strategies (20). In Palestine, the combination of moderate willingness, low uptake, and strong expressed need for more information suggests that health-system communication and policy-level planning may be as important as individual knowledge. Integration of HPV education into reproductive health services, school health programs, and community cancer-awareness initiatives could therefore provide practical entry points for improving informed vaccine decision-making.

This study has several strengths. First, it addresses an important and underexplored public health topic in the Palestinian context, where evidence regarding women's awareness of HPV infection and perceptions of HPV vaccination remains limited. Second, the study provides a multidimensional assessment of HPV-related awareness by examining not only prior awareness and factual knowledge, but also attitudes, self-reported vaccine uptake, acceptability, and perceived barriers to vaccination. This broader analytical approach allows for a more comprehensive understanding of the factors that may influence HPV vaccine decision-making among women. Third, the identification of the discrepancy between low vaccine uptake and comparatively higher willingness to receive the vaccine provides a particularly relevant finding for public health planning, as it suggests the presence of modifiable barriers that may represent potential targets for future interventions.

Several limitations should also be considered when interpreting the findings. The cross-sectional design precludes any inference regarding causality between sociodemographic characteristics, knowledge, attitudes, and vaccine-related behaviors. The study used an open online convenience sampling strategy, which limits representativeness. Women with internet access, higher educational attainment, greater health interest, or stronger engagement with online health-related content may have been more likely to participate. This is reflected in the sample composition, which was skewed toward younger women and those with higher education. Therefore, the findings should not be interpreted as nationally representative estimates of HPV awareness, knowledge, or vaccine acceptability among all women in Palestine. Rather, they provide exploratory evidence on HPV-related knowledge and attitudes among women reached through online recruitment. The study sample was also skewed toward younger and more highly educated women, which may limit the generalizability of the findings to the wider population of women in Palestine. Furthermore, all data were self-reported and therefore may be subject to recall bias or social desirability bias, particularly with respect to vaccination status and attitudes toward sensitive health topics. Finally, although the knowledge score provided a useful measure for assessing factual understanding, the categorization of knowledge levels may not fully capture the complexity of HPV-related health literacy.

Future research should seek to build on these findings using larger and more representative samples drawn from different geographic, social, and educational backgrounds. Longitudinal studies may help clarify how awareness, knowledge, and attitudes toward HPV vaccination evolve over time and whether improved knowledge translates into greater vaccine uptake. In addition, qualitative research could provide deeper insight into the social, cultural, and health-system factors that shape women's perceptions of HPV vaccination and influence decision-making. Further studies are also needed to evaluate the effectiveness of targeted educational interventions, healthcare-provider counseling, and community-based awareness strategies in improving HPV-related knowledge and vaccine acceptance. Such research would be valuable for informing contextually appropriate prevention policies and strengthening future efforts toward HPV control and cervical cancer prevention in Palestine.

## Conclusion

This study found limited awareness and low factual knowledge regarding HPV infection and HPV vaccination among adult women in Palestine, together with low self-reported HPV vaccine uptake. However, willingness to receive the vaccine was higher than actual uptake, suggesting a gap between acceptability and utilization. Lack of awareness and concerns regarding vaccine safety were the most frequently reported barriers, and supportive vaccination attitudes were associated with both higher factual knowledge and vaccine-related outcomes in exploratory analyses. These findings should be interpreted cautiously because of the cross-sectional design and online convenience sampling. Nevertheless, they highlight the need for culturally appropriate HPV education, stronger healthcare-provider counseling, and improved access to reliable information as potential strategies to support future cervical cancer prevention efforts in Palestine.

## Data Availability

The raw data supporting the conclusions of this article will be made available by the authors, without undue reservation.
