# Supplementary material for: Awareness, factual knowledge, attitudes, and acceptability of human papillomavirus vaccination among adult women in Palestine: a cross-sectional study
Source: Front Glob Womens Health. 2026 Jul 21;7:1841337. doi: 10.3389/fgwh.2026.1841337 (PMC13433610; doi:10.3389/fgwh.2026.1841337)
Supplement: Supplementary file 1 [file Supplementaryfile1.docx]

**Supplementary Table S1A. Subgroup differences in HPV vaccine awareness and higher factual knowledge (N = 424)**

| **Variable** | **Category** | **N** | **Heard of HPV vaccine n (%)** | **p value** | **Higher factual knowledge n (%)** | **p value** |
| --- | --- | --- | --- | --- | --- | --- |
| Age group | 18-24 | 274 | 80 (29.2) | 0.007 | 52 (19.0) | 0.288 |
|  | 25-34 | 55 | 26 (47.3) |  | 13 (23.6) |  |
|  | >=35 | 95 | 40 (42.1) |  | 25 (26.3) |  |
| Marital status | Single | 279 | 85 (30.5) | 0.017 | 54 (19.4) | 0.191 |
|  | Ever married | 145 | 61 (42.1) |  | 36 (24.8) |  |
| Educational level | Secondary or less | 48 | 8 (16.7) | 0.006 | 9 (18.8) | 0.656 |
|  | Higher education | 376 | 138 (36.7) |  | 81 (21.5) |  |
| Employment status | Not employed | 242 | 73 (30.2) | 0.033 | 47 (19.4) | 0.295 |
|  | Employed | 182 | 73 (40.1) |  | 43 (23.6) |  |
| Monthly family income | Low | 39 | 10 (25.6) | 0.070 | 5 (12.8) | 0.074 |
|  | Average | 232 | 73 (31.5) |  | 44 (19.0) |  |
|  | High | 153 | 63 (41.2) |  | 41 (26.8) |  |

*Note.* P values were calculated using the chi-square test or Fisher's exact test, as appropriate. Higher factual knowledge was defined as >=7 correct responses on the 12-item scale.

**Supplementary Table S1B. Subgroup differences in HPV vaccine uptake and willingness to receive HPV vaccination (N = 424)**

| **Variable** | **Category** | **N** | **Received HPV vaccine n (%)** | **p value** | **Willing to receive HPV vaccine n (%)** | **p value** |
| --- | --- | --- | --- | --- | --- | --- |
| Age group | 18-24 | 274 | 36 (13.1) | 0.125 | 152 (55.5) | 0.795 |
|  | 25-34 | 55 | 11 (20.0) |  | 32 (58.2) |  |
|  | >=35 | 95 | 8 (8.4) |  | 50 (52.6) |  |
| Marital status | Single | 279 | 40 (14.3) | 0.246 | 159 (57.0) | 0.301 |
|  | Ever married | 145 | 15 (10.3) |  | 75 (51.7) |  |
| Educational level | Secondary or less | 48 | 5 (10.4) | 0.576 | 26 (54.2) | 0.880 |
|  | Higher education | 376 | 50 (13.3) |  | 208 (55.3) |  |
| Employment status | Not employed | 242 | 27 (11.2) | 0.200 | 128 (52.9) | 0.273 |
|  | Employed | 182 | 28 (15.4) |  | 106 (58.2) |  |
| Monthly family income | Low | 39 | 7 (17.9) | 0.062 | 25 (64.1) | 0.350 |
|  | Average | 232 | 22 (9.5) |  | 130 (56.0) |  |
|  | High | 153 | 26 (17.0) |  | 79 (51.6) |  |

*Note.* P values were calculated using the chi-square test or Fisher's exact test, as appropriate. Willingness in this descriptive subgroup table is shown for all participants; the adjusted willingness model in Table 7 was restricted to unvaccinated women.
